# Integrative approach detects natural hybridization of sympatric lambaris species and emergence of infertile hybrids

**DOI:** 10.1038/s41598-019-40856-4

**Published:** 2019-03-13

**Authors:** Ana Paula Barbosa Pinheiro, Rafael Magno Costa Melo, Daniel Fonseca Teixeira, José Luís Olivan Birindelli, Daniel Cardoso Carvalho, Elizete Rizzo

**Affiliations:** 1 Departamento de Morfologia, Instituto de Ciências Biológicas, Universidade Federal de Minas Gerais, UFMG, Belo Horizonte, C. P. 486, 31270-901 Minas Gerais, Brazil; 2 Programa de Pós-graduação em Biologia de Vertebrados, Pontifícia Universidade Católica de Minas Gerais, PUC Minas, Belo Horizonte, 30535-610 Minas Gerais, Brazil; 3 Departamento de Biologia Animal e Vegetal, Universidade Estadual de Londrina, UEL, Londrina, C. P. 10.011, 86057-970 Paraná, Brazil

## Abstract

Despite its relevance for ecology, evolution and conservation of species, natural hybridization and hybrids biology are still poorly studied in freshwater fish. Here, we tested the hypothesis that sympatric species *Astyanax paranae* and *A*. *fasciatus* are able to interbreed in the natural environment and presented evidence for the first record of hybridization between these species. We analyzed anatomical traits, gametogenesis, reproductive biology, and genetic variations of the COI and S7 genes of both species and putative hybrids. Intermediate morphometric and meristic features were observed in hybrids when compared to *A*. *paranae* and *A*. *fasciatus*. Overlap in reproductive season was showed for these species, with greater reproductive activity from August to January, but hybrids did not present any sign of gonadal maturation. Oogonia and perinucleolar follicles as well as spermatogonia and primary spermatocytes were found in hybrids, but previtellogenic and vitellogenic follicles, spermatids, and spermatozoa were absent. Moreover, several alterations in gametogenesis were detected, such as interrupted meiosis in both males and females, vacuolated and degenerated germ cells, increased interstitial tissue, and presence of immune cells. Molecular analyses supported the hypothesis of hybridization between *A*. *paranae* and *A*. *fasciatus*. Overall, our multidisciplinary approach also provides strong evidence that hybrids are infertile.

## Introduction

Hybridization consists of the crossing of groups or individuals that differ genetically, including crosses between lineages of the same species - intraspecific or between individuals of different species – interspecific^1^. In general, the hybridization is considered more common in plants than animals^2^ but is more frequent in fish than in other vertebrates^3^. In freshwater fish, hybridization is favored by ecophysiological attributes, such as external fertilization, competition for spawning territories, poor mechanisms of reproductive isolation, unequal abundance of the parental species, susceptibility to contact between individuals who have recently diverged, as well as cohabitation in limited environments^3–5^.

Although natural hybridization is an event considered rare historically, it is relatively common, especially in groups such as fish and amphibians^3,6^. Natural hybridization is also related to the evolution of species, since the transfer of DNA from one species to the genomic set of another species by repeated backcrossing between hybrids and parental species (introgressive hybridization) may constitute a considerable source of genetic variability in populations^7^. It is estimated that at least 10% of the animal species can undergo natural interspecific hybridization^8^.

In general, interspecific hybrids have intermediate morphological characteristics compared to parental species^3^ and have historically been identified by morphometric and meristic analyses^4^. With the advancement of molecular biology techniques, identification of hybrids has been based on detection of genetic polymorphism in regions of the nuclear and mitochondrial DNA^9–11^. In this context, DNA barcoding using the COI gene has been widely used in molecular taxonomy for fish identification^12,13^ and may be also an auxiliary tool for the identification of hybrids as well as other mitochondrial genes^4,14–16^. In addition, the S7 ribosomal protein gene has been successfully used in order to confirm the parental contributions in hybridization studies^17,18^.

Interspecific hybridization is generally facilitated by phylogenetic proximity between the species, since they tend to have less genetic divergence and a similar diploid chromosome number or genome size^19,20^. However, cross-breeding often leads to the formation of individuals containing extra chromosomes, which do not have homologous pairs and, then, do not allow normal meiotic cell division at the time of gamete formation^1^. Therefore, many hybrids may present gonadal alterations and sterility, characteristics rarely investigated in hybridization studies.

The *Astyanax* genus of the family Characidae is one of the most diverse freshwater taxa of the Neotropical ichthyofauna^21^. These fish are popularly known as lambaris and are geographically distributed from Argentina to the border region between Mexico and the United States^22^. *Astyanax paranae* Eigenmann, 1914 is typically a headwaters species, while *A*. *fasciatus* (Cuvier, 1819) is a more generalist species and can be found in a wide range of aquatic systems, including lentic and lotic environments^23,24^. *Astyanax* species usually present a long reproductive period with multiple spawning, external fertilization, and absence of parental care^24–26^. Although they are small fish, the lambaris have ecological and commercial importance, since they are forage species for several predators^27^ and are caught in professional and artisanal fishing, possibly constituting protein source for riverine populations^24^. In addition, *Astyanax* species have been indicated as sentinels for environmental investigations of pollutants^28,29^.

Due to their morphofunctional plasticity, lambaris have a wide geographic distribution, and it is common to find different species coexisting in sympatry in some habitats. Sympatry may lead to ecological and reproductive niche overlapping, eventually favoring interbreeding between related species^19^. Considering the phylogenetic proximity and morphophysiological similarities found among fish from the genus *Astyanax*, it is possible that the overlap of niches culminates in occasional cross-fertilization of the gametes, in a process of hybridization. In this regard, evidence of interbreeding between populations of *Astyanax bimaculatus* from Paraná and São Francisco River basins after deviation of a tributary was reported^20^.

Recent studies on hybridization of freshwater fish species are mainly based on cytogenetic and molecular approaches, with identification of hybrids resulting from artificial crossing in aquaculture, introduction of species, and damage to habitats^9,11,16,20,30^. The aim of this study was to test, using a multi-disciplinary approach, the hypothesis that the sympatric species *Astyanax paranae* and *A*. *fasciatus* are able to interbreed in the natural environment. We analyzed anatomical traits, gametogenesis, reproductive biology, and genetic variations of COI and S7 genes of both species and its putative hybrids.

## Methods

### Study area and fish sampling

The study was performed in an area of waterfall (22°13′7.04″S 44°32′23.14″W) at the headwaters of the Grande River, southeastern Brazil. The area is close to Itatiaia National Park, an important conservation unit of the Atlantic Forest biome, which has little anthropic influence and preserved ciliary forest.

Quarterly samplings during a reproductive cycle were carried out. A total of 494 lambaris specimens were collected using gillnets (0.8, 1.3, and 1.5 cm between opposing knots). The specimens presented three different morphotypes: the species *A*. *paranae* (n = 239; MZUEL18646) and *A*. *fasciatus* (n = 212; MZUEL18645) and the morphotype *Astyanax* sp. – putative hybrids (n = 43; MZUEL18647), which exhibited anatomical features of both *A*. *paranae* and *A*. *fascitaus*. The fish were identified according to Graça and Pavanelli^31^ and deposited at the Museum of Zoology of the State University of Londrina (MZUEL). During fish collection, physicochemical parameters of the water were obtained in each period using a Horiba U-51 multiparameter probe.

In the field, fish caught alive were euthanized by immersion in eugenol 85 mg/L, and then the biometric data of each specimen were obtained. All the indicated procedures followed the principles established by the Brazilian College of Animal Experimentation (COBEA). The study was approved by the State Forestry Institute (IEF - fishing license no. 153) and the Ethics Committee on Animal Use of the Federal University of Minas Gerais, Brazil (CEUA-UFMG, protocol no. 92).

### Morphometric and meristic analyses

A total of 21 specimens were examined, seven each of *A*. *paranae*, *A*. *fasciatus*, and putative hybrids. All specimens were collected and preserved together. Counts and measurements followed Fink and Weitzman^32^, except for longitudinal scale rows above lateral line, counted from the middorsal scale row to lateral line, not including the scale of the middorsal row immediately anterior to dorsal-fin origin or the small scale on dorsal-fin base; and scale rows below lateral line, counted from lateral line to pelvic-fin origin, the half scale was only counted when the scale immediately anterior to pelvic-fin origin had at least half height above pelvic-fin insertion. Pored scales of lateral line included pored scales on base of caudal-fin rays. Differences in body shape among samples were determined by analyzing the morphological traits through principal component analysis (PCA) using PAST software^33,34^. Data were log transformed prior to analysis.

### Biological indexes and fecundity

Total length (TL), body weight (BW), and gonads weight (GW) were obtained using a caliper with accuracy of 0.01 cm and an analytical balance, accurate to 0.01 g. These biometric data were used to calculate the biological indices: gonadosomatic (GSI = 100 GW/BW) and Fulton condition factor (K = 100 BW/TL³). The stages of gonadal maturation were determined based on macroscopic and microscopic characteristics of the gonads and GSI variations^28^: 1, resting; 2/3, ripening/ripe and 4, spawning.

To estimate fecundity, samples from the middle region of mature ovaries were collected during the reproductive season peak (January). The samples (n = 20 *A*. *paranae* and 20 *A*. *fasciatus*) were weighed and fixed in Gilson’s fluid (100 ml 60% of ethyl alcohol, 880 ml of distilled water, 15 ml of 80% nitric acid, 18 ml of glacial acetic acid and 20 g of mercuric chloride). The dissociated vitellogenic oocytes were separated and counted under a stereoscopic microscope. The number of oocytes per gram of ovary was determined and used to calculate batch fecundity based on total ovary weight. Relative fecundity was estimated from the number of vitellogenic oocytes in the ovaries per unit of gonadal weight (GW).

### Light and transmission electron microscopy

For histological analyses of gametogenesis and gonadal maturation, samples from the middle region of the gonads of each fish were fixed in Bouin’s fluid (75 ml of saturated picric acid solution 1.3% in water, 25 ml of formalin and 5 ml of glacial acetic acid). Gonad samples were dehydrated in crescent concentrations of ethanol (70, 80, 95 and 100%), embedded in paraffin, sectioned at 5 μm thickness, and stained with hematoxylin-eosin (HE) and Gomori trichrome.

For ultrastructure analysis of the germ cells of putative hybrids, samples of the gonads were fixed in modified Karnovsky’s solution (2.5% glutaraldehyde and 2% paraformaldehyde). At the Microscopy Center of the Federal University of Minas Gerais, the samples were submitted to secondary fixation in 2% osmium tetroxide and were incubated in sucrose solution with 4% uranyl acetate overnight. Then, they were dehydrated in ethanol and embedded in Epon resin. The ultrathin sections were examined using a transmission electron microscope at 120 kV (Tecnai G2–12 – Spirit Biotwin FEI).

### Morphometry of the germ cells

The diameter of the germ cells in each developmental phase was measured in each species using Axiovison image analysis software coupled to a photomicroscope. Type A and B oogonia (G_A_ and G_B_), early and advanced perinucleolar (PN_1_ and PN_2_), previtellogenic (PV, with cortical alveoli) and vitellogenic oocytes (V) were measured at 100 or 1000 x magnification from five fish in the ripening/ripe stage of each species, totaling 50 measurements of the germ cells in each developmental phase. The follicular and theca cell layers were not considered for these measurements. Similarly, the nuclear diameter of 50 male germ cells (G_A_ and G_B_, types A and B spermatogonia, SP_1_ and SP_2_, primary and secondary spermatocytes; T, spermatids and Z, spermatozoa) from five fish in the ripening/ripe stage of each species was measured at 1000 x magnification. Germ cells were identified according to Quagio-Grassiotto *et al*.^35^, Lubzens *et al*.^36^ and Schultz *et al*.^37^.

To determine the proportion (%) of the germ cells, 10 images of the ovaries and testes of each group (*A*. *paranae*, *A*. *fasciatus* and putative hybrids) in resting stage were randomly chosen and analyzed with Image J software. Using a grid with 550 intersections, germ cells in different developmental phases^35–37^ and the following gonadal components were quantified: tubular lumen, interstitial tissue, inflammatory infiltrate, blood vessel, tissue degeneration, and apoptotic body. Myoid and Leydig cells were included in the interstitial tissue. The sex of the putative hybrids was recognized by histological analyses of the gonads, i.e. presence of at least one oocyte and absence of cyst-like structures for females, and presence of tubular organization and cyst-like structures for males.

### Immunofluorescence

To investigate whether germ cells in putative hybrids were compromised with apoptosis, we performed immunofluorescence for caspase 3. Gonad samples were fixed in 4% paraformaldehyde solution, embedded in paraffin, sectioned at 5 μm thickness, and submitted to an immunocytochemistry reaction using primary antibody rabbit anti-caspase-3 (polyclonal). For antigen recovery, sections were boiled in 10 mM sodium citrate buffer at pH 6.0 for 20 min and incubated with 2% BSA solution (bovine serum albumin) in PBS (phosphate-buffered saline) for 30 min to block non-specific reactions. Next, primary antibody (dilution 1: 100) was applied to the sections overnight in a humidified chamber at 4 °C. Sections were washed in PBS and then incubated with secondary antibody anti-rabbit ALEXA 488 (1: 500). Nuclear DNA labelling was performed using 4,6-diamidino-2-phenyl-indole (DAPI, 1:500). For negative control, the treatment with the primary antibody was omitted. Sections were examined with a fluorescence microscope (Axio Imager Z2 – ApoTome 2 Zeiss) from the Microscopy Center of the Federal University of Minas Gerais.

### Molecular analysis

For mitochondrial Cytochrome Oxidase Subunit I (COI) gene, DNA was obtained from caudal fin tissue fixed in absolute ethanol from 26 *A*. *paranae*, 25 *A*. *fasciatus*, and 23 putative hybrids. DNA extraction was carried out using the salt extraction method, adapted from Aljanabi and Martinez^38^. The sequences were amplified using M13-tailed primer cocktails for the COI gene^39^. The amplification was done using the PCR technique in a thermocycler using 10 μl of a solution composed of 7 μl of ultrapure water, 0.3 μl of dNTP (10 mM), 1 μl of buffer MgCl_2_, 0.4 μl of M13-tailed primer (10 μM), 0.3 μl of Taq DNA polymerase (5 U/μl), and 1 μl of template DNA. The PCR conditions used for amplification of the COI gene were: denaturation at 95 °C (2 min), then 35 denaturation cycles at 94 °C (30 s), primer annealing at 56 °C (30 s), extension at 72 °C (1 min), and final extension at 72 °C (10 min). PCR products were bidirectionally sequenced by the Sanger method using an Applied Biosystems, 3500 Genetic Analyzer and a commercial BigDye® Terminator v3.1 sequencing kit. Sequences were compared to reference sequences from GenBank (http://www.ncbi.nlm.nih.gov): JN 988740 and JN 988739 for *A*. *paranae*, and JQ 353543 and JQ 353534 for *A*. *fasciatus*.

For nuclear S7 ribosomal protein gene (first intron), DNA was amplified using the primers S7RPEX1F and S7RPEX2R^40^, and the following PCR conditions: initial denaturation at 94 °C (2 min) followed by 35 denaturation cycles at 94 °C (45 s), primer annealing at 59 °C (45 s), extension at 72 °C (45 s), then final extension at 72 °C (7 min). PCR products were sequenced using an Applied Biosystems, 3730 Genetic Analyzer. Samples of caudal fin tissue from eight specimens of each morphotype (*A*. *paranae*, *A*. *fasciatus* and putative hybrids) were used for this analysis.

For the construction of the consensus of COI sequences (contigs), DNA Baser software version 4.16 was used. The alignment of the contigs was done in MEGA 6.0, followed by manual conference and editing. PopART 1.7 was used to construct the haplotype network. Kimura 2-parameter distance (K2P) was calculated using the software DnaSP 5.10.01. For the analysis of the S7 gene, the sequences were aligned using the Geneious R8 software.

### Statistical analysis

Statistical analyses were performed using GraphPad Prism software version 5.0 for Windows. Values are expressed as means ± SD or SEM, and the results were considered significant at a 95% confidence interval. As the biological data did not show a normal distribution, they were analyzed using the Kruskal-Wallis test (followed by Dunn’s post-test) and Mann-Whitney test (when applicable).

## Results

To verify if the water quality was favorable for fish reproduction, we evaluated its physicochemical parameters which were summarized in the Supplementary Table S1. Low temperatures throughout the year and high levels of dissolved oxygen were obtained. The amount of total dissolved solids was according resolution no. 357 of National Council for the Environment, CONAMA^41^, Brazil, indicating water quality suitable for supporting fish reproduction.

### Morphometric and meristic data

Sampled specimens of *A*. *paranae* and *A*. *fasciatus* were identified based on several meristic characteristics, including the number of scales on the lateral line, scale rows around caudal peduncle, scale rows above and below lateral line, and the number of branched anal-fin rays (see Supplementary Table S2). These meristic traits were intermediate in putative hybrids. For example, these specimens had 18–19 branched anal-fin rays, whereas *A*. *paranae* had 14 to 15 and *A*. *fasciatus* had 22 to 25. The body shape of putative hybrids was also intermediate between *A*. *paranae* and *A*. *fasciatus* (see Supplementary Table S3). The major differences in the body shape of *A*. *paranae* and *A*. *fasciatus* were related to the caudal peduncle length, anal-fin base length, and horizontal eye diameter.

**Figure 1:**
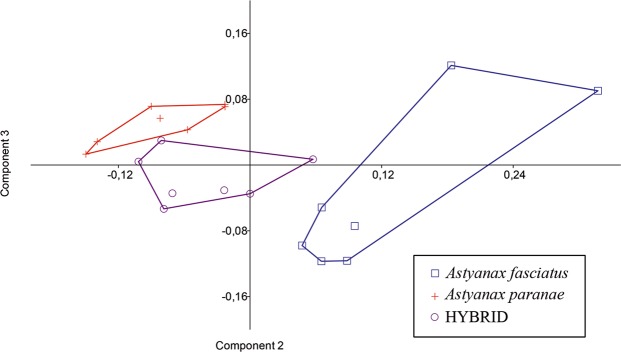
Dispersion diagram of individual scores in samples of *Astyanax fasciatus*, *A*. *paranae* and hybrids in the space defined by principal components 2 and 3.

In the PCA analysis, PC1 accounted for 70% of the observed variation, but included only positive values, as PC1 represents variation in size of specimens. PC2 and PC3 accounted for 16.2% and 6.2% of the found variation, respectively, and included positive and negative values, representing most of the body shape variation of the samples (see Supplementary Table S4). In the diagram from the PCA analysis, *A*. *paranae* and *A*. *fasciatus* were found to be completely non-overlapping groups on the PC2 axis with the hybrid population included between these species (Fig. 1). The major variables in PC 2 were: caudal peduncle length and caudal peduncle depth for *A*. *paranae*, and anal-fin base length and horizontal eye diameter for *A*. *fasciatus*.

### Biometrical data and biological indexes

The body size of the lambaris varied from 7.6 to 14.2 cm TL, and from 6.52 to 38.90 g BW. In general, specimens of *A*. *fasciatus* were bigger than *A*. *paranae* (Table 1). Sexual dimorphism in size was observed between females and males of *A*. *paranae* and *A*. *fasciatus* (Mann-Whitney test). The Fulton condition factor (K) was statistically higher for females and males of *A*. *paranae* when compared to *A*. *fasciatus* (p < 0.0001), while females of hybrids showed an intermediate value between both species of lambaris. Hybrids specimens did not show gonadal maturation and exhibited very thin gonads and the lowest values of GSI among the compared fish (Table 1). In general, higher GSI was observed in females than males, and the highest values occurred in *A*. *paranae* when compared to *A*. *fasciatus* and hybrids.

**Table 1:** Biometric data and biological indexes of *A*. *paranae*, *A*. *fasciatus* and hybrids from the headwaters of Grande River.

	Females			Males		
	*A*. *paranae*	Hybrids	*A*. *fasciatus*	*A*. *paranae*	Hybrids	*A*. *fasciatus*
TL (cm)	9.68 ± 0.06^a^	10.23 ± 0.46^ab^	10.92 ± 0.08^b^	9.03 ± 0.15^a^	10.94 ± 0.23^bc^	10.46 ± 0.37^c^
BW (g)	12.93 ± 0.27^a^	14.24 ± 2.27^ab^	15.97 ± 0.37^b^	9.77 ± 0.51^a^	17.98 ± 1.26^b^	12.93 ± 1.39^a^
GSI	6.93 ± 0.55^a^	0.07 ± 0.02^b^	2.71 ± 0.37^c^	2.09 ± 0.61^a^	0.10 ± 0.04^b^	0.32 ± 0.12^ab^
K	1.41 ± 0.01^a^	1.25 ± 0.04^bc^	1.20 ± 0.01^c^	1.31 ± 0.02^a^	1.32 ± 0.02^a^	1.15 ± 0.02^b^

Values represent mean ± SEM. In a line, different letters indicate significant differences between individuals of the same sex, p < 0.05, Kruskal-Wallis, Dunn’s post-test. (TL) total length, (BW) body weight, (GSI) gonadosomatic index, (K) Fulton condition factor.

### Reproductive biology

**Figure 2:**
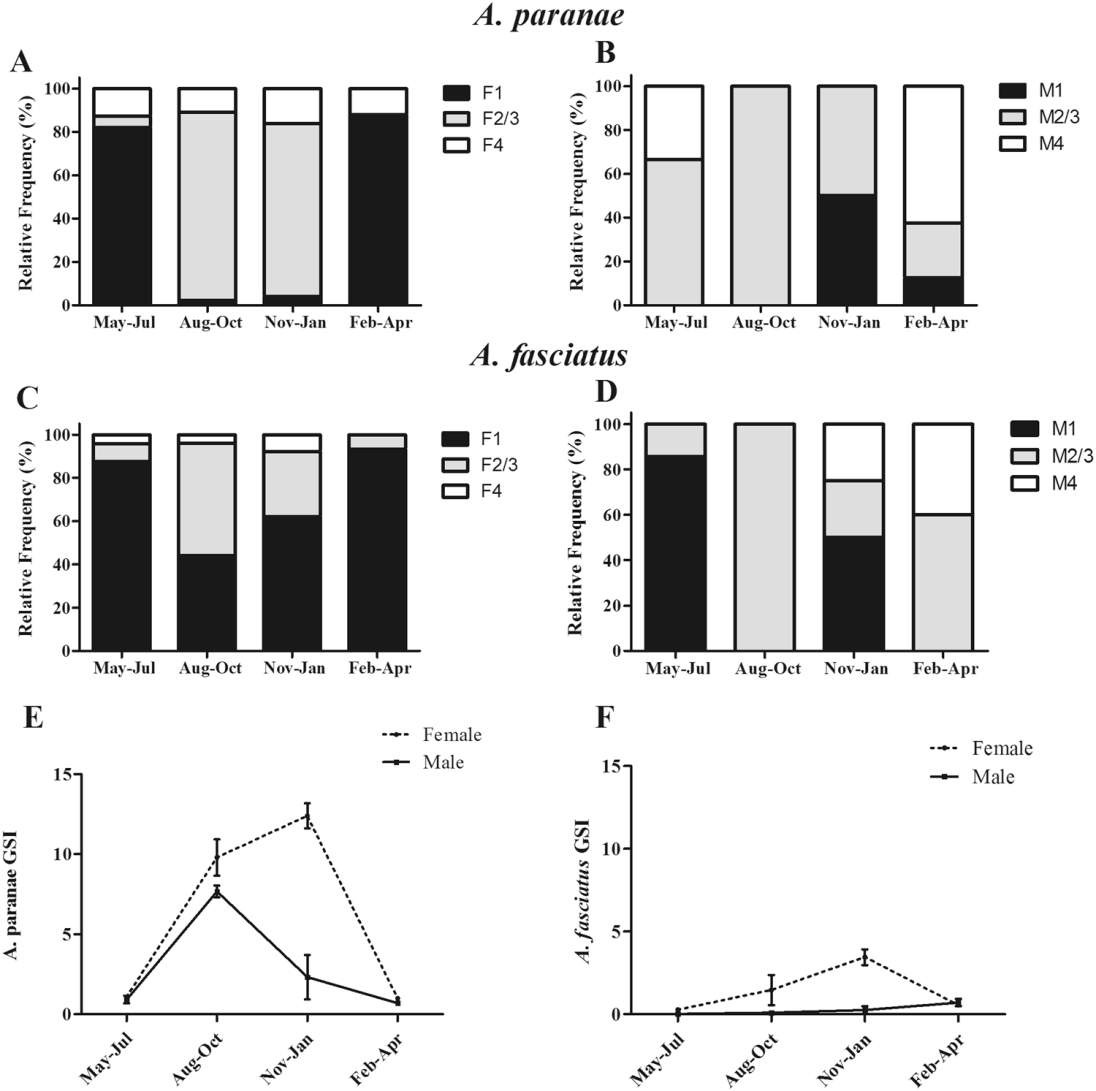
Reproductive parameters of *A*. *paranae* and *A*. *fasciatus* from the Grande River headwaters. (**A–D**): Seasonal distribution of the relative frequencies (%) of the gonad maturation stages for females and males of *A*. *paranae* (**A**,**B**) and *A*. *fasciatus* (**C**,**D**). Gonad maturation stages: (1) resting, (2/3) maturation/ripe and (4) spawned. (**E**,**F**) Seasonal distribution of the gonadosomatic index (GSI) for females and males of *A*. *paranae* (**E**) and *A*. *fasciatus* (**F**). Values represent mean ± SEM. Sample size is given above bars. *A*. *paranae*: n = 217 females and 22 males. *A*. *fasciatus*: n = 195 females and 17 males.

To confirm whether *A*. *paranae* and *A*. *fasciatus* reproduce in the headwaters of the Grande River, we comparatively assessed gametogenesis and gonadal maturation (see Supplementary Fig. S1). The relative frequency of gonadal maturation stages revealed specimens in the ripening/ripe stage in most sampling periods, with a higher frequency in the August–October and November–January quarters (Fig. 2A–D), when females had the higher GSI (Fig. 2E,F). Spawned females were also found in almost all periods, thus indicating a prolonged reproductive season with greater reproductive activity from August to January for *A*. *paranae* and *A*. *fasciatus* in the headwaters of the Grande River. Females in the resting stage were more frequent in *A*. *fasciatus* than *A*. *paranae* from August to January. Batch fecundity (BF) was not significantly different between *A*. *paranae* and *A*. *fasciatus* (p = 0.335) (see Supplementary Table S5).

### Morphological alterations in gonads of hybrids

**Figure 3:**
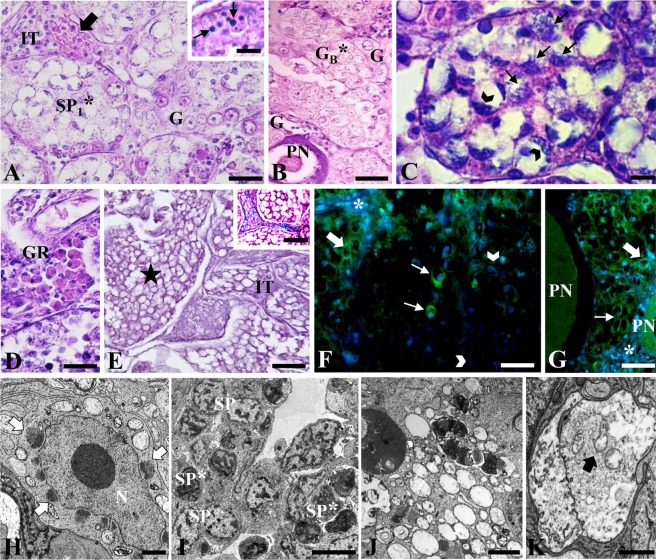
Histological sections of gonads of hybrids stained with hematoxylin and eosin (**A**–**E**) and Gomori trichrome (detail **E**), immunofluorescence for caspase 3 (**F**,**G**) and electron microscopy (**H–K**). A: testis with spermatocytes in degeneration (SP_1_*), interstitial tissue (IT), blood vessel (arrow) and spermatogonia (**G**). Detail: Apoptotic bodies. (**B**) Ovary with a normal perinucleolar oocyte (PN), type B oogonia in degeneration (G_B_*) and normal oogonia (**G**). (**C**) Spermatocytes in degeneration showing chromatin attached to the nuclear envelope at the periphery of the cell (arrowhead), little evident nucleolus and signs of interrupted meiosis with formation of chromosomes and altered spindle fibers (arrows). (**D**) Presence of granulocytes (GR) in the interstitial tissue. (**E**) Degenerated tissue (star) with cells without evident nucleus and increased interstitial tissue (IT). Detail: same tissue stained with Gomori trichrome. (**F**) Immunolocalization of caspase 3 in testis, positive reaction for spermatocytes in degeneration (fine arrow) and spermatogonia (thick arrow); negative reaction (arrowhead) and interstitial tissue (asterisk). (**G**) Immunolocalization of caspase 3 in ovary, positive reaction for type B oogonia in degeneration (arrow), normal oogonia (arrowhead) and perninucleolar oocytes (PN); negative reaction for interstitial tissue (asterisk). (**H**) Ultrastructure of spermatogonia presenting elongated nucleus (N) and presence of nuages (arrow). (**I**) Normal spermatocytes (SP) and in degeneration (SP*). (**J**) Detail of the cytoplasm of an intensely vacuolated cell. (**K**) Large autophagic vacuole, containing organelles in degeneration (arrow) inside. Bar: (**A**) 20 µm, (detail) 6 µm; (**B**) 40 µm; (**C**,**I**) 5 µm; (**D**) 20 µm; (**E**) 15 µm, (detail) 10 µm; (**F**) 20 µm, detail (6 µm); (**G**) 30 µm; (**H**) 2 µm; (**J**) 0.5 µm, (**K**) 1 µm.

**Figure 4:**
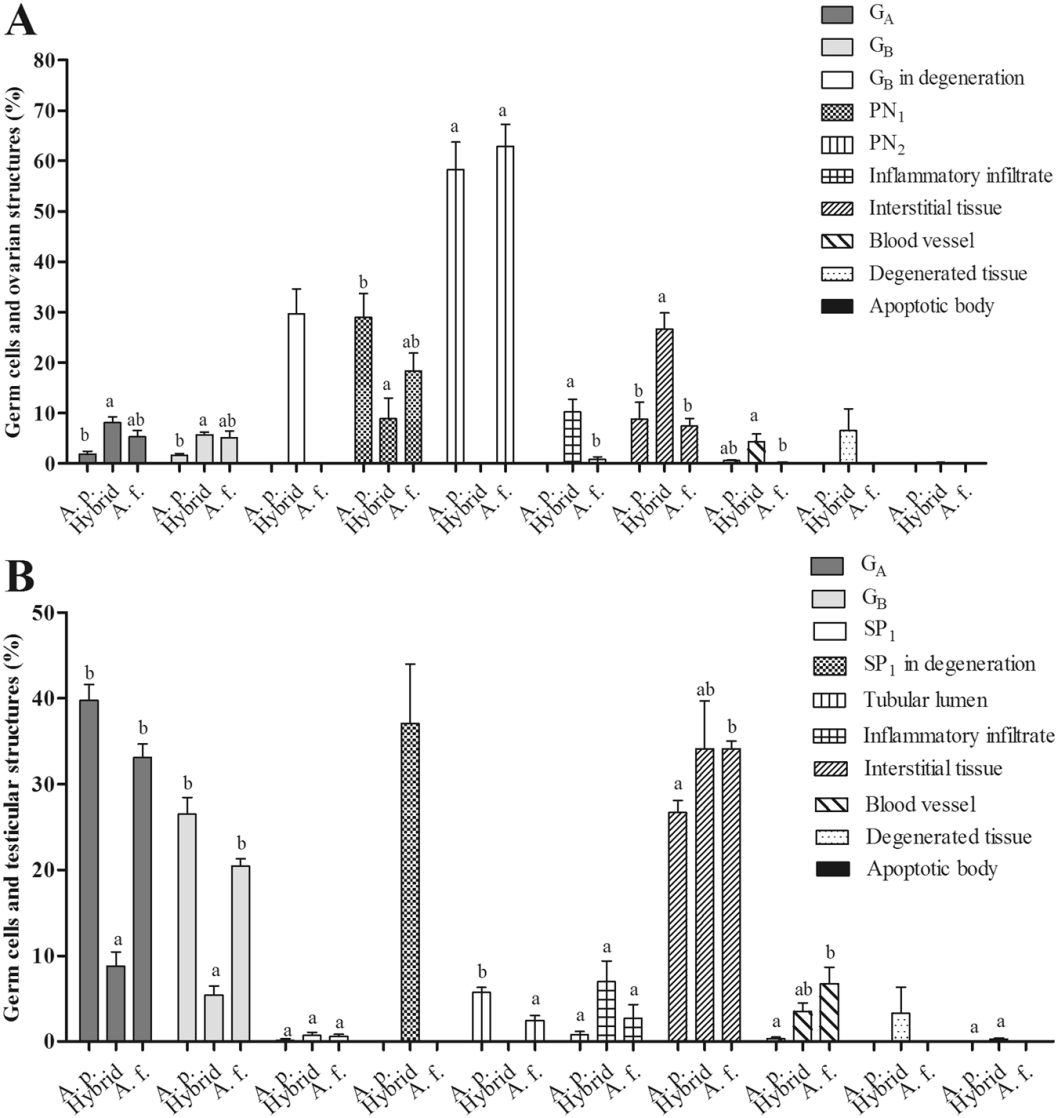
Proportion (%) of germ cells and ovarian (**A**) and testicular (**B**) structures in *A*. *paranae* (A. p.), hybrids and *A*. *fasciatus* (A. f.) from the Grande River headwaters. Values represent mean ± SEM. Different letters indicate significant differences among the groups A. p., hybrid and A. f.

Hybrid specimens did not present gonadal maturation, and morphological analyzes showed several tissue alterations, such as vacuolated and degenerated germ cells, increased interstitial tissue, presence of immune cells, and degeneration of organelles (Fig. 3). We observed alterations in the first meiotic division, and most cells had chromatin aggregates close to nuclear envelope, in a typical pattern of apoptosis (Fig. 3A–C,F–I). In these animals, a continuum was observed from tissues that presented some progression in gametogenesis, exhibiting a perinucleolar oocyte or few spermatogonia and spermatocytes spread between the vacuolated cells (Fig. 3A,B,H,I), until fully vacuolated tissue with totally degenerated germ cells, which did not advance in meiosis (Fig. 3E). However, previtellogenic and vitellogenic oocytes as well as spermatids and spermatozoa were not observed in any of the hybrid specimens. Some immune cells were also observed in the gonads of these animals (Fig. 3D,K). The immunolocalization of caspase 3 occurred in several of degenerated cells (B oogonia in females and primary spermatocytes in males) (Fig. 3F,G). Wide cytoplasmic vacuolization and autophagic vacuoles were also frequently observed in hybrids. (Fig. 4L,M).

### Gametogenesis

Germ cells of *A*. *paranae* and *A*. *fasciatus* had similar diameters in most developmental phases and the gametes formed at the end of this process did not present significant differences (see Supplementary Table S6). The proportion (%) of germ cells in the resting stage (Fig. 4) showed no significant difference in females of *A*. *paranae* and *A*. *fasciatus*. However, the males of *A*. *fasciatus* had increased interstitial tissue (34.13 ± 0.86%) and blood vessels (6.69 ± 1.97%) as well as decreased tubular lumen than males of *A*. *paranae*. In the hybrids, the ovaries had G_A_ (8.11 ± 1.16%), G_B_ (5.64 ± 0.53%), and PN_1_ (8.88 ± 4.09%), besides histopathological alterations such as increased interstitial tissue (26.63 ± 3.18%) and inflammatory infiltrate (10.19 ± 2.54%) when compared to resting females of *A*. *paranae* and *A*. *fasciatus*. The presence of degenerated tissue (6.51 ± 4.35%), apoptotic bodies (0.09 ± 0.06%), and B oogonia in degeneration (29.65 ± 4.93%) were also detected in hybrids, which were all absent in the *A*. *paranae* and *A*. *fasciatus*. Regarding the males, hybrid specimens had intermediate proportion of interstitial tissue and blood vessels when compared to the other two species and a lower amount of G_A_ and G_B_ (8.76 ± 1.70; 5.37 ± 1.08%), as well as the presence of degenerated tissue (3.30 ± 3.03%) and SP_1_ in degeneration (37.01 ± 6.96%), which were absent in *A*. *paranae* and *A*. *fasciatus*.

### Molecular analyses

After alignment, manual conferencing, and trimming of the ambiguous ends, the COI sequences obtained were 564 base pairs in length. A total of 26 COI sequences were obtained from *A*. *paranae*, 25 from *A*. *fasciatus*, and 23 from hybrids.

**Figure 5:**
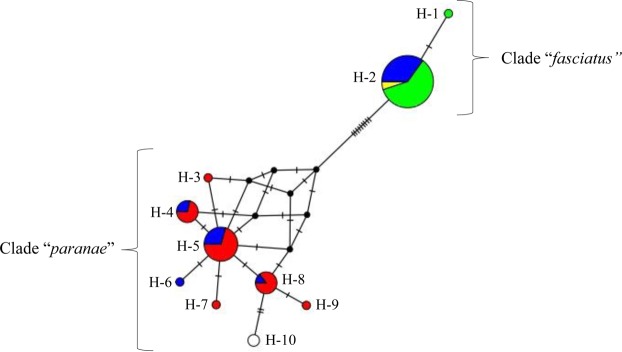
Haplotype network built with COI gene sequences of hybrids (blue), *A*. *fasciatus* (green) and *A*. *paranae* (red) from the Grande River headwaters obtained in the present study and reference sequences of *A*. *fasciatus* (yellow) and *A*. *paranae* (white) downloaded from GenBank.

The haplotype network showed the presence of two phylogenetically distinct clades, one referring to *A*. *paranae* and another referring to *A*. *fasciatus* (Fig. 5). A total of 9 haplotypes was detected in the 74 lambaris samples, and they were numbered from H-1 to H-9 in the haplotype network. The H-10 haplotype was found only in reference sequences for the *A*. *paranae* species obtained from the GenBank. Among the haplotypes sampled in this study, two were related to *A*. *fasciatus* clade (H-1 and H-2) and the other seven to *A*. *paranae* clade (H-3, H-4, H-5, H-6, H-7, H-8, and H-9). The H-2 haplotype was the most representative in this study, being shared by 40 animals, including most of the *A*. *fasciatus* specimens. In *A*. *paranae*, the most representative haplotype was H-5, which was shared by 15 individuals. The H-6 haplotype was observed in a specimen of the morphotype *Astyanax* sp. - hybrids, although according to the BOLD database, it belongs to the *A*. *paranae* clade. The haplotype network showed that *A*. *paranae* and *A*. *fasciatus* did not share haplotypes, while hybrids had shared haplotypes of both species of lambaris (H-2, H-4, H-5, H-6, and H-8), even though only showing one morphotype. Seven specimens of hybrids were grouped with *A*. *paranae* (Hybrid clade *paranae*) while 16 specimens were grouped with *A*. *fasciatus* (Hybrid clade *fasciatus*).

The mean genetic distance (K2P) between the sequences of the mitochondrial COI gene ranged from 0 to 2.2% (see Supplementary Table S7). The genetic distance analysis confirmed that the *A*. *paranae* and *A*. *fasciatus* morphotypes correspond to distinct species, since the distance between these two groups was 2.2%. In addition, the observed divergences between samples and reference sequences were low (0.5% for *A*. *paranae* and 0% for *A*. *fasciatus*). The genetic distance found between *A*. *paranae* haplotypes was 0.171%, while *A*. *fasciatus* haplotypes were 0.014% distant. Thus, a DNA “barcode gap” was observed among species of the genus *Astyanax*, showing that DNA barcoding was able to distinguish these species successfully.

**Figure 6:**
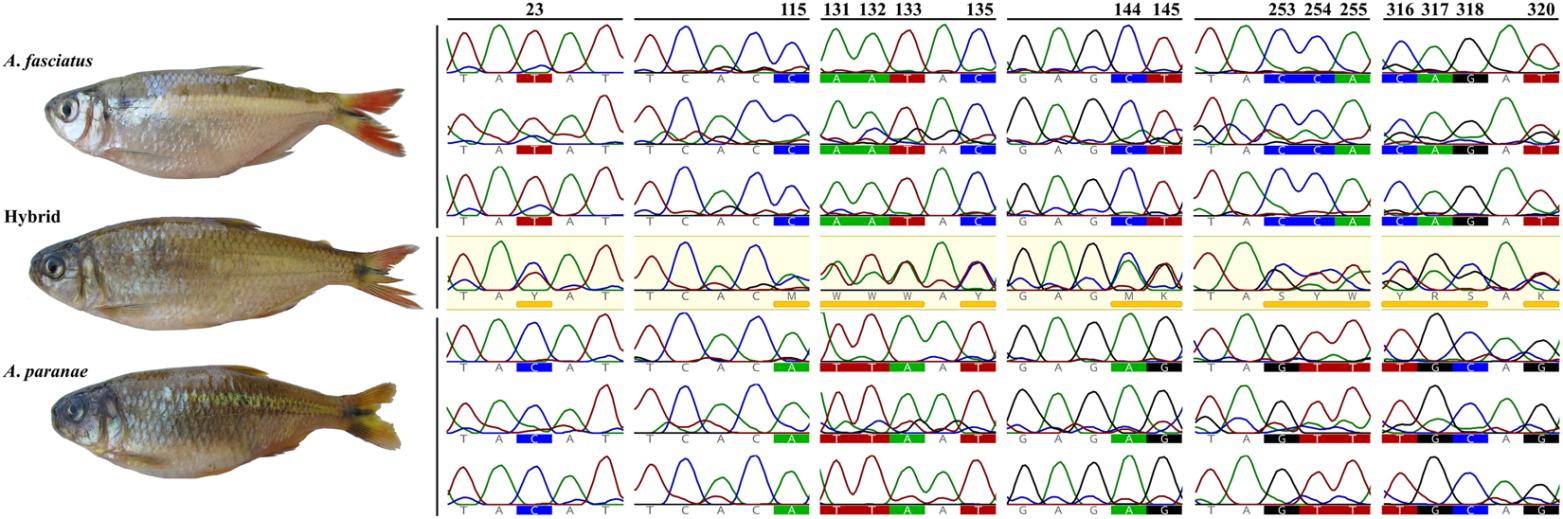
Photographs of lambaris from the Grande River headwaters and genetic alignment of the nuclear S7 gene fragment. Chromatograms show the hybrid individual’s heterozygous peaks (underlined with yellow bars) at the diagnostic sites, where *A*. *fasciatus* and *A*. *paranae* present fixed differences.

The S7 sequences obtained were 390 base pairs in length after alignment, manual conferencing, and trimming of the ambiguous ends. A total of 196 variable sites were observed in hybrids when compared to *A*. *paranae* and *A*. *fasciatus*. Hybrid specimens showed well-marked double peaks in their DNA sequencing chromatograms, an evidence of being heterozygotes^42,43^, with an allele inherited from each parental species. *A*. *paranae* and *A*. *fasciatus* presented fixed differences between each other at these variable sites (Fig. 6).

## Discussion

In South America, reduction in the abundance and distribution of species, removal of isolation barriers of populations, river transposition, and escape of domestic species from fish farming contribute to hybridization and are an increasing threat to fish fauna^9,11,16,20,30^. Here, we report for the first time the natural hybridization between the lambaris *A*. *paranae* and *A*. *fasciatus* by using a multidisciplinary approach.

Morphological analysis of putative hybrids showed intermediate anatomical features when compared to *A*. *paranae* and *A*. *fasciatus*, as expected for hybrid fish^3,4^. Among these features, it includes branched anal-fin rays, which is one of the main features used to diagnose *A*. *paranae* and closely related species, the so-called *Astyanax scabripinnis* species complex^44^. In Brazilian aquaculture, the common artificial crossing between the catfishes *Pseudoplatystoma corruscans* and *P*. *reticulatum* gives rise to a hybrid that shows a morphological characteristic with an intermediate pattern of its lateral body spots in relation to parental species^45^.

In the current study, both species showed sexual dimorphism in size, with larger and heavier females than males, thus corroborating previous studies in *Astyanax* species^46,47^. The presence of small specimens of *A*. *paranae* and *A*. *fasciatus* in reproductive activity in this study indicates that first sexual maturation occurs early in the development of lambaris. Indeed, Ferreira^46^ found specimens of *A*. *paranae* reproducing with a size of 3–5 cm in the Mogi Guaçu River basin. Although the hybrids exhibit from 8.6 to 14.2 cm, a length at which most lambaris of *A*. *paranae* and *A*. *fasciatus* species shows reproductive activity, these specimens did not present gonadal maturation. Also, the trend of greater weight presented by the hybrids in relation to the other species may be due to the absence of energy investment in reproduction^1^.

The frequency distribution of the gonadal maturation stages described in this study demonstrated the overlap of reproductive period between *A*. *paranae* and *A*. *fasciatus* species. In *A*. *paranae*, spawned females were observed throughout all collections, suggesting that this species reproduces throughout the year in the headwaters of the Grande River, showing multiple spawns. This same reproductive strategy has been associated with *A*. *fasciatus* in some studies^24,48^. In addition, the morphology of ovaries and testes was similar in *A*. *paranae* and *A*. *fasciatus*, as also observed in *A*. *bimaculatus*, *A*. *scabripinnis*, and *A*. *fasciatus* from the Velhas River^49^. Morphological and reproductive similarities between the species studied reflects the phylogenetic proximity and the overlap of reproductive niches (area and/or period of spawning), features known to favor the hybridization^3,8,19,20,30,50^. In fact, a confined environment, such as the waterfall lagoon inhabited by the lambaris species of the present study, has been considered favorable for accidental fertilization of eggs of another species^51^.

Histological and ultrastructural analyses performed on the gonads of the hybrids strongly suggest that the first phase of meiosis was interrupted in these fish. Apparently, it did not advance to stages after prophase due to the incorrect paring of chromosomes and formation of altered spindle fibers in B oogonia and primary spermatocytes, leading to the alterations observed in their gonads. Evidence of this process is that spermatids, spermatozoa, and previtellogenic and vitellogenic follicles were not found in any of the hybrid specimens. Chromatin aggregates attached to the nuclear envelope and positive reaction for caspase 3 indicated apoptosis of the germ cells, which was triggered after meiosis failure. Apoptosis can be a common finding in germ cells of hybrids and it was observed in 45% of seminiferous tubules analyzed in mules (crossing of male *Equus asinus* and female *Equus caballus*)^52^. Along with the morphological alterations observed in this study, degenerated tissue was formed and immune cells engulfed tissue remnants, while there was a remodeling for the formation of connective interstitial tissue. Despite the above-mentioned changes, some normal germ cells were observed in the hybrids, showing that a few cells advanced in meiosis similar to that found in sunfish hybrids and mules^52,53^. The ultrastructural analyses showed degeneration of organelles and presence of autophagic vacuoles, with autophagy possibly being related to nutrient recycling to sustain cell metabolism during tissue degeneration. Similarly, hybrids of cichlid fish showed increased interstitial tissue, degenerated cells containing large lysosomes, and apoptotic nuclei^54^.

During meiosis, recognition and paring of homologous chromosomes occur with the formation of the synaptonemal complex and crossing over, with the establishment of chiasmata in prophase in a highly regulated process^55^. Failures during this and other stages of meiosis have been associated with human infertility^56,57^. Here, we believe that failure in meiosis of hybrids triggered the morphological damage observed in these fish, i.e. tissue degeneration and apoptosis of B oogonia and primary spermatocytes. In triploid individuals, the three homologues align side by side during meiosis, but only two chromosomes are involved in the synapse^58^, and this can lead to the interruption of meiosis^59^. In medaka hybrids, the meiotic cell cycle was interrupted before reaching metaphase I, and when hybrids of both sexes were cultured together, females were able to spawn but the eggs did not develop, and males did not produce functional spermatozoa^60^. Also, defects in meiosis in sunfish hybrids led to sterility and production of altered gametes^53^.

COI analysis identified *A*. *paranae* and *A*. *fasciatus* as two phylogenetically distinct clades and the divergence between the haplotypes was greater than 2%, which is considered a cut-off limit for distinct fish species^61^. The proximity of the standard reference value found in this study (2.2%) may be related to the phylogenetic proximity between the analyzed species, a feature correlated to hybridization^30^. In fact, Rossini *et al*.^13^ suggest that *A*. *paranae* and *A*. *fasciatus* present recent radiation with low values of genetic distance. Although these species are close related, they present different chromosome counts in their karyotypes (*A*. *paranae* 2n = 50 and *A*. *fasciatus* 2n = 46–48)^62–64^. Therefore, the interrupted meiosis and sterility of the hybrids found in the present study are probably related to the presence of extra chromosomes, which possibly do not have homologous pairs, preventing the progression of meiosis and, consequently, formation of gametes in these specimens^1^.

The fact that a single morphotype shares haplotypes of two distinct species may be an indication of hybridization, since haplotypes have uniparental inheritance^65^. Thus, the specimens of morphotype *Astyanax* sp. may be considered hybrids resulting from the crossing between the lambaris species *A*. *paranae* and *A*. *fasciatus*. Considering that COI is a mitochondrial gene and its inheritance is maternal, this crossing could be bidirectional: females of *A*. *paranae* with males of *A*. *fasciatus* or males of *A*. *paranae* with females of *A*. *fasciatus*, since the inheritance was associated with both species.

Corroborating the COI data, analysis of S7 nuclear gene evidenced the hybridization process, since all the specimens of *Astyanax* sp. morphotype were heterozygous and showed several sites of double peaks within their DNA sequencing chromatograms, a peak from each pure parental species. Moreover, all variation found within the S7 fragment of the hybrids analyzed in this study was limited to the variability presented by *A*. *fasciatus* and *A*. *paranae*. In order to confirm a hybridization process, an ideal genetic marker cannot show variation within a species, besides presenting fixed differences between parental species and potentially showing hybrids as heterozygous^43^. In this sense, microsatellites often fail in diagnosing hybridization between closely related species, because they show wide range in allele size and, consequently, overlap during analysis^43^. Therefore, S7 gene first intron region proved to be an effective marker for hybridization diagnosis between the species *A*. *paranae* and *A*. *fasciatus*.

In the current study, genetic data supported the hypothesis of natural hybridization between *A*. *paranae* and *A*. *fasciatus*. Furthermore, we highlight the data of the reproductive biology and germ cells morphology that strongly corroborate the molecular data and, in addition, suggest that hybrids are infertile. Thus, integrating data on anatomy, reproductive biology, gonadal morphology, and mitochondrial and nuclear DNA, the present study provides strong evidence of natural hybridization between two species of lambaris. Assessment of hybridization markers, including morphological and genetic issues, is of great relevance to improve the knowledge on conservation status of the phylogenetically close species that exhibit reproductive niche overlap in natural environments.

## Supplementary information

Supplementary Information Pinheiro et al., 2019

## Data Availability

The datasets generated and/or analyzed during the current study are available from the corresponding author on reasonable request. The sequences obtained in this study were deposited in GenBank under accession numbers MH626656-MH626729 and MK318200-MK318216.
