# Supplementary material for: Integrative approach detects natural hybridization of sympatric lambaris species and emergence of infertile hybrids
Source: Sci Rep. 2019 Mar 13;9:4333. doi: 10.1038/s41598-019-40856-4 (PMC6416303; doi:10.1038/s41598-019-40856-4)
Supplement: Supplementary file 1 — Supplementary Information Pinheiro et al., 2019 [file 41598_2019_40856_MOESM1_ESM.pdf]

Supplementary Information for

Integrative approach detects natural hybridization of sympatric lambaris species and emergence of infertile hybrids

Ana Paula Barbosa Pinheiro[1], Rafael Magno Costa Melo[1], Daniel Fonseca Teixeira[2], José Luís Olivan Birindelli[3], Daniel Cardoso Carvalho[2], Elizete Rizzo[1*]

[1]Departamento de Morfologia, Instituto de Ciências Biológicas, Universidade Federal de Minas Gerais, UFMG, Belo Horizonte, C. P. 486, 31270-901, Minas Gerais, Brasil.

[2]Programa de Pós-graduação em Biologia de Vertebrados, Pontifícia Universidade Católica de Minas Gerais, PUC Minas, Belo Horizonte, 30535-610, Minas Gerais, Brasil.

[3]Departamento de Biologia Animal e Vegetal, Universidade Estadual de Londrina, UEL, Londrina, C. P. 10.011, 86057-970, Paraná, Brasil.

[*]To whom correspondence should be addressed. Email: ictio@icb.ufmg.br

Department of Morphology, Institute of Biological Sciences, Federal University of Minas Gerais, Av. Antônio Carlos, 6627, Belo Horizonte, Minas Gerais, Brazil PO Box 486, 31270-901. Phone +55 31 34092819.

Table S1: Water quality in the headwaters of Grande River

	May-July	August- October	November-January	February-April
Temperature (°C)	13.54 ± 0.55	11.26 ± 0.01	16.18 ± 0.01	14.02 ± 0.01
pH	6.57 ± 0.06	6.07 ± 0.03	5.98 ± 0.01	6.54 ± 0.01
Dissolved oxygen (mg/L)	13.28 ± 0.27	12.64 ± 0.05	11.19 ± 0.07	12.75 ± 0.27
Conductivity (µS/cm)	12.00 ± 0.01	12.00 ± 0.01	12.33 ± 0,33	12.00 ± 0.01
Total suspended solids (mg/L)	8.00 ± 0.01	8.00 ± 0.01	8.00 ± 0.01	8.00 ± 0.01

Data (mean ± SEM) were obtained during a reproductive cycle.

Table S2. Meristic data of samples of *Astyanax fasciatus*, *A. paranae* and interspecific hybrids.

**Characters**	***A. fasciatus*** (n=7) Range			***A. paranae*** (n=7) Range			**Hybrids** (n=7) Range		
Pored scales on lateral line*	39	-	42	35	-	38	36	-	38
Scale rows between dorsal-fin origin and lateral line*	7	-	8	6	-	6	6	-	7
Scales rows between lateral line and pelvic-fin origin*	6	-	6	5	-	5	5	-	6
Pre-dorsal scales	11	-	13	11	-	13	11	-	12
Scale rows around caudal peduncle*	16	-	16	14	-	14	14	-	16
Dorsal-fin branched rays	9	-	9	9	-	9	9	-	9
Anal-fin branched rays*	22	-	25	14	-	15	18	-	19
Pectoral-fin branched rays	13	-	15	12	-	14	12	-	137
Pelvic-fin branched rays	7	-	7	6	-	6	7	-	8
Upper caudal-fin branched rays	8	-	9	9	-	9	9	-	9
Lower caudal-fin branched rays	8	-	8	8	-	8	8	-	8
Outer premaxillary teeth	3	-	5	3	-	4	4	-	4
Inner premaxillary teeth	5	-	5	5	-	5	5	-	5
Maxillary teeth	1	-	1	1	-	2	1	-	2
Dentary teeth	5	-	5	5	-	6	5	-	6

Asterisks indicate most important characters.

Table S3. Morphometric data of samples of *Astyanax fasciatus*. *A. paranae* and interspecific hybrids.

Characters	*Astyanax fasciatus*						*Astyanax paranae*						Hybrids					
	n	Mean	Range			SD	n	Mean	Range			SD	n	Mean	Range			SD
Standard Length (mm)	7	80.75	74.56	-	88.90		7	83.66	74.86	-	92.22		7	72.56	62.6	-	94.05	
**Percentages of SL**																		
Depth at dorsal-fin origin	7	33.61	30.42	-	36.56	1.99	7	30.85	29.08	-	32.25	1.06	7	32.24	30.66	-	35.05	1.63
Snout to dorsal-fin origin	7	48.71	45.99	-	50.15	1.42	7	50.49	48.68	-	53.41	1.66	7	49.84	48.42	-	51.28	1.09
Snout to pectoral-fin origin	7	25.17	21.58	-	26.89	1.71	7	26.88	25.98	-	27.64	0.61	7	26.36	24.49	-	29.71	1.66
Snout to pelvic-fin origin	7	46.39	44.94	-	48.58	1.42	7	49.60	48.57	-	50.47	0.62	7	49.38	48.03	-	51.42	1.36
Snout to anal-fin origin	7	64.95	62.74	-	66.58	1.28	7	67.80	66.54	-	68.78	0.97	7	67.35	66.18	-	69.09	1.11
Caudal-peduncle depth	7	10.56	8.02	-	11.42	1.15	7	11.86	11.35	-	12.14	0.31	7	11.51	11.20	-	12.42	0.44
Caudal peduncle length*	7	11.52	7.24	-	12.41	1.89	7	14.88	12.73	-	16.34	1.30	7	12.88	12.03	-	13.58	0.61
Pectoral-fin length	7	18.40	14.62	-	20.11	1.76	7	18.05	16.67	-	19.91	1.07	7	18.56	16.14	-	19.25	1.11
Pelvic-fin length	7	14.70	10.54	-	15.89	1.86	7	14.39	14.05	-	14.87	0.28	7	14.76	13.62	-	15.76	0.68
Dorsal-fin base length	7	13.40	10.39	-	14.42	1.41	7	13.95	12.61	-	15.04	1.01	7	13.53	12.81	-	14.05	0.45
Dorsal-fin depth	7	23.45	19.76	-	25.19	1.83	7	21.74	20.89	-	22.42	0.65	7	23.30	21.38	-	24.52	1.26
Anal-fin base length*	7	26.44	25.06	-	28.26	1.13	7	20.47	19.86	-	20.88	0.41	7	23.71	22.12	-	25.51	1.07
Anal-fin lobe length	7	15.06	13.01	-	16.83	1.20	7	14.67	13.17	-	16.77	1.23	7	15.55	14.29	-	16.45	0.93
Eye to dorsal-fin origin	7	35.58	32.55	-	37.00	1.44	7	39.13	36.52	-	41.21	1.45	7	37.51	36.17	-	39.17	1.07
Dorsal-fin origin to caudal-fin base	7	53.67	51.26	-	55.40	1.66	7	50.65	45.68	-	55.51	3.77	7	53.72	51.82	-	54.82	1.04
Bony head length	7	24.38	23.80	-	25.05	0.49	7	25.51	23.15	-	27.31	1.75	7	25.69	25.43	-	26.68	0.45
**Percentages of head length**																		
Horizontal eye diameter*	7	35.59	30.69	-	38.23	2.61	7	27.36	25.19	-	29.37	1.63	7	30.45	27.49	-	32.81	1.80
Snout length	7	27.83	25.54	-	30.81	1.89	7	29.58	27.28	-	32.88	2.35	7	28.93	27.61	-	29.66	0.90
Least interorbital distance	7	33.29	31.40	-	35.40	1.72	7	36.22	31.94	-	40.61	3.36	7	33.36	31.20	-	35.85	2.06
Upper jaw length	7	44.12	41.66	-	46.92	2.18	7	45.47	41.19	-	50.35	3.55	7	42.18	40.72	-	44.11	1.07

Asterisks indicate most important characters.

Table S4. Coefficients of variables on the first, second and third principal components of traits studied for *Astyanax fasciatus*, *A. paranae* and interspecific hybrids.

Characters	PC 1	PC 2	PC 3
Standard Length	0.19311	0.10844	0.10243
Depth at dorsal-fin origin	0.18713	0.18481	-0.12594
Snout to dorsal-fin origin	0.20909	0.043068	0.095554
Snout to pectoral-fin origin	0.2091	-0.055709	-0.033929
Snout to pelvic-fin origin	0.20911	0.043799	0.11973
Snout to anal-fin origin	0.20912	0.062206	0.098139
Caudal-peduncle depth*	0.20913	-0.31074	-0.15377
Caudal peduncle length*	0.20914	-0.57773	-0.14631
Pectoral-fin length	0.20915	0.062166	-0.21205
Pelvic-fin length	0.20916	-0.19054	-0.46304
Dorsal-fin base length	0.20917	0.042309	0.065869
Dorsal-fin depth	0.20918	0.15205	-0.19407
Anal-fin base length*	0.20919	0.48252	-0.25787
Anal-fin lobe length	0.2092	0.20052	-0.054426
Eye to dorsal-fin origin	0.20921	0.0075885	0.17393
Dorsal-fin origin to caudal-fin base	0.20922	0.16614	0.029744
Bony head length	0.20923	0.031913	0.18535
Horizontal eye diameter*	0.20924	0.3752	-0.24098
Snout length	0.20925	0.035377	0.41589
Least interorbital distance	0.20926	-0.006654	0.39942
Upper jaw length	0.20927	0.057524	0.25564
**% variation**	**70.35**	**16.16**	**6.12**

Asterisks indicate most important variables.

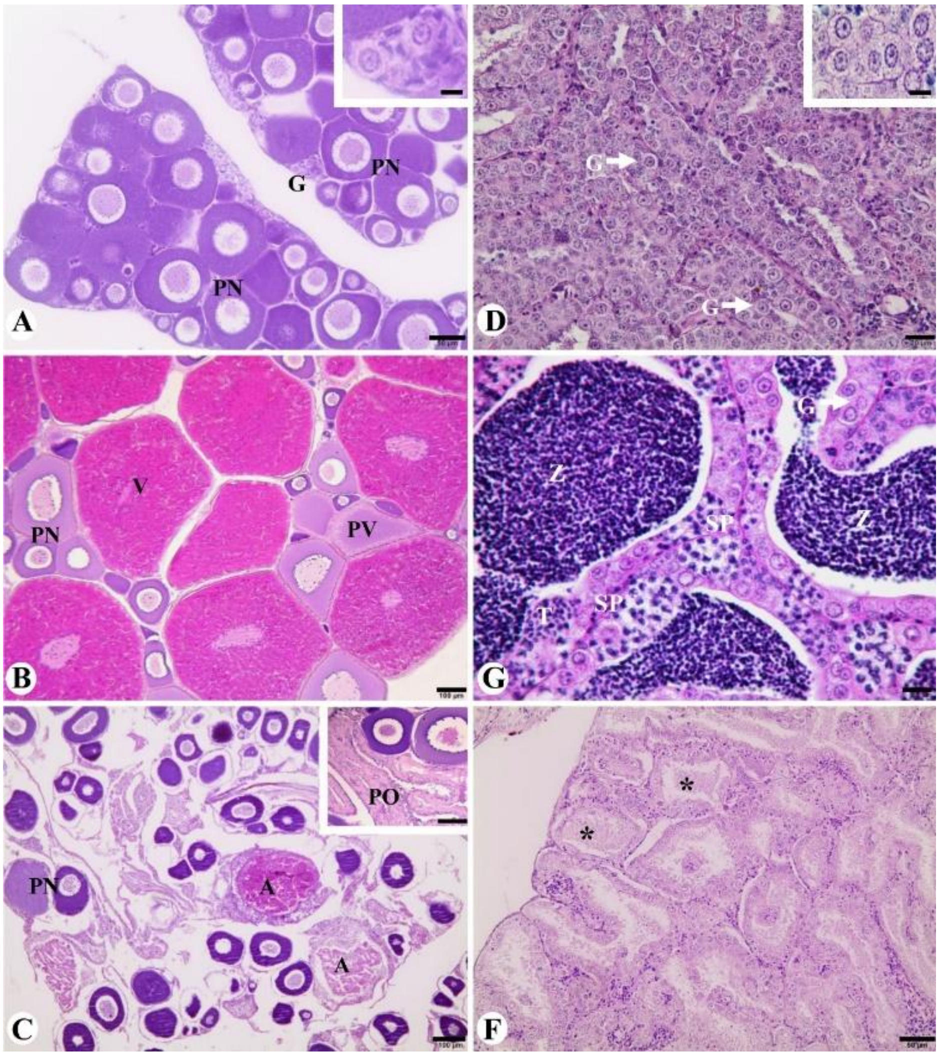

Figure S1. Histological sections of ovaries (A, B, C) and testes (D, E, F) of lambaris *A. paranae* and *A. fasciatus* in different gonad maturation stages stained with hematoxylin-eosin. A: Resting (F1) with ovigerous lamellae containing perinucleolar (PN) follicles and ovogonia (G) nests. B: Ripening/ripe (F2/3) with presence of perinucleolar (PN), pre-vitellogenic (PV) and vitellogenic (V) follicles. C: Spawned (F4) with atretic (A) and post-ovulatory (PO) follicles (detail) in regression and increased connective tissue between follicles. D: (M1) seminiferous tubules showing closed lumens and numerous spermatogonia (detail). E: (M2/3) cysts of germ cells in different developmental stages: spermatogonia (G), spermatocytes (SP), spermatids (T) and abundant spermatozoa (Z). F: (M4) seminiferous tubules (asterisks) with empty lumen or containing residual spermatozoa. Bar: (A) = 100 µm, detail = 7 µm; (B) = 130 µm; (C) = 170 µm, detail = 110 µm; (D) = 17 µm, detail = 7 µm; (E) = 13 µm; (F) = 30 µm.

Table S5: Batch fecundity of *A. paranae* e *A. fasciatus* from the headwaters of Grande River.

Species	Batch Fecundity		Relative Fecundity			
	Range	Mean	TL (cm)	BW (g)	GW (g)	r (GW)
A. paranae	2439-10217	5582.82 ± 413.04[a]	575.55 ± 38.23[a]	372.40 ± 21.49[a]	1984.16 ± 81.91[a]	0.848*
A. fasciatus	2676-8120	5668.57 ± 440.61[a]	525.94 ± 41.06[a]	325.51 ± 24.88[a]	2662.84 ± 150.80[b]	0.669*

Values represent mean ± SEM (µm). In a column, different letters indicate significant differences among the species. Pearson (r) correlation coefficient between batch fecundity and total length (TL), body weight (BW) and gonadal weight (GW). Asterisks indicate significant correlation between batch fecundity and GW.

Table S6: Diameters of female germ cells and nucleus of male germ cells in *A. paranae* e *A. fasciatus* captured on headwaters of the Grande River.

Females			Males		
	A. paranae	*A. fasciatus*		*A. paranae*	*A. fasciatus*
**G_A**	14.08 ± 0.29[a]	14.57 ± 0.33[a]	**G_A**	6.37 ± 0.10[a]	6.70 ± 0.12[a]
**G_B**	10.83 ± 0.32[a]	11.92 ± 0.33[b]	**G_B**	4.62 ±0.07[a]	4.80 ± 0.08[a]
**PN_1**	133.60 ± 1.89[a]	146.70 ± 2.95[b]	**SP_1**	3.70 ± 0.05[a]	3.96 ± 0.05[b]
**PN_2**	207.90 ± 4.49[a]	229.90 ± 5.83[b]	**SP_2**	2.29 ± 0.04[a]	2.44 ± 0.03[b]
**PV**	324.10 ± 5.48[a]	341.90 ± 5.17[b]	**T**	1.93 ± 0.02[a]	1.87 ± 0.02[b]
**V**	609.20 ± 5.90[a]	598.80 ± 7.20[a]	**Z**	1.84 ± 0.01[a]	1.81 ± 0.01[a]

Values represent mean ± SEM (µm). In a line, different letters indicate significant differences among the species, considering the same sex, $p < 0.05$, Mann-Whitney. (G_A) and (G_B): type A and type B oogonia or spermatogonia; (PN_1 and PN_2) initial and advanced perinucleolar oocytes, (PV) pre-vitellogenic and (V) vitellogenic oocytes; (SP_1 and SP_2) primary and secondary spermatocytes; (T) spermatids; (Z) spermatozoa.

Table S7: Mean genetic distance (K2P) for the COI gene (%) between the different lambari morphotypes captured on headwaters of Grande River and reference sequences.

**Morphotypes**	**1**	**2**	**3**	**4**	**5**	**6**
1. *A. paranae*	-					
2. Hybrid clade *paranae*	0.2	-				
3. Hybrid clade *fasciatus*	2.2	2.2	-			
4. *A. fasciatus*	2.2	2.2	0.0	-		
5. Reference *A. paranae*	0.5	0.6	2.5	2.5	-	
6. Reference *A. fasciatus*	2.2	2.2	0.0	0.0	2.5	-
